# Supplementary material for: Empirical methods for controlling false positives and estimating confidence in ChIP-Seq peaks
Source: BMC Bioinformatics. 2008 Dec 5;9:523. doi: 10.1186/1471-2105-9-523 (PMC2628906; doi:10.1186/1471-2105-9-523)
Supplement: Additional File 1 — A variety of html documents from the USeq web site detailing the available applications, their best usage, output file type descriptions, command line menus, etc. [file 1471-2105-9-523-S1.zip › USeqUserGuides/JavaDoc.zip!/JavaDoc/bioroot/antibody/package-frame.html]

bioroot.antibody


bioroot.antibody

Classes 
  
AntibodyBase
  
AntibodyBean
  
AntibodyModify
  
AntibodyPrep
  
AntibodySpreadSheet
  
AntibodyViewPreferences
  
NewAntibody
  
UseDilution
